# An examination of the nature of localized molecular orbitals and their value in understanding various phenomena that occur in organic chemistry

**DOI:** 10.1007/s00894-018-3880-8

**Published:** 2018-12-26

**Authors:** James J. P. Stewart

**Affiliations:** Stewart Computational Chemistry, 15210 Paddington Circle, Colorado Springs, CO 80921 USA

**Keywords:** Canonical molecular orbitals, Eigenvectors, Localized molecular orbitals, Lewis structures, Delocalization, Rabbit ears, Banana bonds, Reactions, Solids

## Abstract

While canonical molecular orbitals have been used in computational chemistry for almost a century, the use of localized molecular orbitals is relatively new, and generating them has been difficult until recently. This has impeded their routine use in modeling chemical systems and reactions so that, even though localized molecular orbitals can now be generated easily, their usefulness in interpreting chemical phenomena has not been properly appreciated. Localized molecular orbitals can provide useful insights into chemical phenomena such as two-electron bonds, π delocalization, and lone pairs. A potentially important application would be interpreting the phenomena that occur in chemical reactions, in particular those reactions which can be described using the Lewis curly-arrow electron pushing convention. This paper considers how canonical and localized molecular orbitals are generated, their usefulness and limitations, and some issues that could be considered controversial regarding their nature, and it presents examples of the usefulness of LMOs in describing six chemical systems and one reaction.

## Introduction

Quantum theoretical calculations have produced many kinds of results that have been extremely useful for predicting the properties of chemical systems. But while some calculables, such as total energies and forces acting on atoms, have experimental equivalents and are usually referred to as observables, other calculables, in particular molecular orbitals, have no possible experimental equivalent and are commonly referred to as non-observables. Although there is currently no generally accepted definition of observables and non-observables in chemistry [1], from a theoretical perspective the defining characteristic of an observable calculable is that it is the result of an operator acting on a system’s state wavefunction, *Ψ*, in contrast to those quantities that involve only the wavefunction of an individual electron or a pair of electrons, *ψ*_*i*_. Although these latter quantities are calculable and have real expectation values, they have no experimental equivalent, so comparison with any observable property of a real system is impossible. Nevertheless, in describing and interpreting chemical phenomena, especially those involved in reactivity and reactions, the use of calculated non-observables such as individual molecular orbitals has undoubtedly been of considerable value.

In quantum chemistry, there are two common ways of representing the set of occupied molecular orbitals (MOs). One consists of the eigenvectors of the self-consistent field (SCF) Fock matrix; these are the canonical molecular orbitals or CMOs. The other is composed of localized molecular orbitals or LMOs. LMOs are normally localized on one or two atoms with only a small intensity on other nearby atoms, with the important exception that, when delocalized π-systems are present, a LMO might have significant intensity on three or even more atoms. Both sets of MOs consist of orthonormal functions, and each set can be converted into the other by a unitary transform [2], so both representations yield the same electron density and are therefore physically equivalent.

CMOs and LMOs are generated in two very different ways. Conventional quantum chemistry methods use matrix algebra, and these methods produce CMOs when an exact diagonalization of the SCF secular determinant matrix is performed. Recently, Lewis structures have been used in an SCF method [3] that avoids the use of matrix algebra; this method produces LMOs by default. Although both the CMO and the LMO approaches yield the same results for observables such as charge distribution, dipole moment, heat of formation, geometry, etc., they give very different results for non-observables, such as the energies and shapes of the molecular orbitals.

The objectives of this report are to describe some properties of LMOs and to show that they can be useful in describing organic chemical systems and their reactions. To minimize the need for caveats, qualifications, and other distractions that would be necessary if a completely general description of LMOs were to be given, the assumption that the systems involved are normal organic compounds should be made when reading the following discussion. Specifically, they should be nontrivial (not isolated atoms, ions, etc.), devoid of high symmetry of the type that might cause symmetry-related phenomena that would be irrelevant to this discussion, and they should not involve unusual bonding of the type found in non-innocent ligands [4] in organometallic chemistry.

The discussion applies to NDDO-type [5] semiempirical methods only; while it might be applicable to other methods, this should not be assumed. Molecular orbitals generated by methods based on the NDDO approximations are normalized so that the sum of the squares of atomic orbital coefficients is unity. They also form an orthogonal set in that the sum of the products of atomic orbital coefficients for two different MOs is zero. Together, these two properties greatly simplify working with semiempirical molecular orbitals. Finally, all MOs in the occupied set should be regarded as being doubly occupied; that is, fractional occupancy, thermal population, radicals, and other exotica are ignored.

## Matrix algebra methods

The earliest quantum chemical methods used matrix algebra, beginning in 1931 when Hückel [6] published his π-electron model for the electronic configuration of benzene. In this model, a matrix of interaction energies between the 2*p* π orbitals is constructed; when the matrix is diagonalized, the result is a set of eigenvectors and eigenvalues: the CMOs. The Hückel framework, based on matrix algebra, was used as the foundation of many subsequent theoretical chemistry methods, from the ω technique [7] to the Pariser–Parr–Pople method [8, 9], the CNDO and NDDO methods [5], INDO [10], MNDO (the first of the modern semiempirical methods) [11, 12] and its successors AM1 [13], PM3 [14, 15], PM6 [16], RM1 [17], AM1* [18–22], and PM7 [23], as well as a plethora of Hartree–Fock ab initio methods for solving Schrödinger’s equation for chemical systems. Matrix algebra methods have thus proven to be both very successful and very popular for many decades.

From a practical perspective, a severe drawback of using matrix algebra in quantum chemistry methods is that some of the mathematical operations necessarily scale as the cube of the size of the system. This is particularly evident for semiempirical methods when large systems such as biomacromolecules are modeled, as the computational effort for all other operations becomes insignificant compared to matrix operations such as matrix inversion, multiplication, and diagonalization.

## Localized molecular orbital methods

A simple and elegant way of describing the electronic structure of a molecule, in particular organic compounds, is to use Lewis structure [24] diagrams. Published in 1916, Lewis structures are one of the oldest ways of describing molecular electronic structures. These structures have the advantages of being easy to generate and informative. They are very useful for predicting certain qualitative properties, such as the geometric environment of an atom and the type of reactions a molecule might participate in. By their nature, Lewis structures are not quantitative, and as such the development of Lewis structure theory is limited. In 1996, the MOZYME [3] method was published. This method uses Lewis structures as the foundation for a LMO quantitative chemical modeling method.

To lay the groundwork for a discussion of MOZYME, a brief description of Lewis structures will now be given.

### Lewis structures

Lewis structures are generated using only the topology of the system and the number of valence electrons on each atom. Structural elements consist of bonds between atoms, with one line indicating a single bond, two lines a double bond, and three lines a triple bond, and pairs of dots to indicate lone pairs on an atom. Other common symbols used in Lewis structures are a plus sign on an atom to indicate a cationic site and a minus sign to indicate an anionic site. Less common symbols include a single dot on an atom to represent an unpaired electron, an arrow in place of a line to indicate that both electrons in the bond came from the same atom, and a dotted line to represent a fractional covalent bond.

A simple test of the correctness of a Lewis structure is to verify that the expected number of valence electrons on each atom can be worked out from the Lewis structure diagram. Lines represent two-electron covalent bonds, with each bond contributing one valence electron to each of the two atoms it connects. Lone pairs on an atom contribute two valence electrons to that atom, and a negative sign on an atom represents one extra valence electron.

In addition to providing a simple representation of molecular electronic structure, Lewis structures have proven to be very useful for describing reaction mechanisms, where curly arrows [25] are used to show the movement of electrons when chemical bonds are made or broken.

Although Lewis structure theory is only qualitative, it does provide a simple and powerful tool for describing the electronic structures of compounds and for predicting and explaining reaction mechanisms.

### The MOZYME method

Because all Lewis structures involve only local (monatomic or diatomic) interactions, a quantum chemistry model based on Lewis structures should, in principle, be able to avoid the need for matrix algebra, thus reducing the computational effort involved in solving the SCF equations. This model would use the simple Lewis structure as the starting point for constructing a set of localized molecular orbitals for use in a SCF calculation.

Transitioning from Lewis structures to LMOs involves a paradigm shift. Lewis structures involve bonds, lone pairs, charges, etc. These quantities are observables (at least in principle) that can be generated from a set of LMOs. Individual LMOs are two-electron wavefunctions, i.e., non-observables, and while it is both possible and useful to make a one-to-one mapping of Lewis structural elements and LMOs, to equate them would be incorrect or even dangerous as it could easily lead to theoretically nonsensical territory, such as attempting to find the best form of MO to produce a specific bond.

Using LMOs to solve the SCF equations is the principle behind the MOZYME [3] method. A necessary and sufficient condition for a SCF to exist is that all interaction energies between occupied and virtual MOs must vanish. Solving the self-consistent field equations therefore consists of calculating and then annihilating all such interactions.

The MOZYME procedure begins by converting the conventional *s*-*p* and *s*-*p*-*d* orbital basis set on an atom into an equivalent number of hybrid atomic orbitals oriented towards the nearby atoms. These hybrids are then used to construct Lewis-type LMOs, i.e., occupied LMOs that are completely localized on one or two atoms. At the same time, a second set of empty LMOs is constructed. These represent cationic sites, virtual lone pairs, and antibonding diatomic bonds. Together, the two sets form a complete set of orthonormal MOs. However, there are nonzero interactions between the occupied and virtual LMOs, and therefore, when it is first generated, the complete set does not satisfy the SCF conditions. The LMOs are similar to the CMOs at this point in that, at the start of the conventional SCF procedure, the CMOs also have nonzero occupied–virtual interactions.

From this point on, the MOZYME procedure differs from conventional SCF methods in that the secular equations, instead of being constructed using atomic orbital basis functions, are constructed using the LMOs. During the first few iterations of the procedure to solve the SCF equations, the LMO size increases rapidly as annihilation of the occupied–virtual LMO interactions causes the LMOs to expand onto nearby atoms. In subsequent iterations, this expansion slows down and, for large systems, stops when each LMO contains between 50 and 200 atoms. Even when the LMOs are this large, most occupied and virtual LMOs for macromolecular systems still have no atoms in common, and in such cases the interaction is automatically zero. For large systems, this means that the number of elements in the Hamiltonian that need to be annihilated scales approximately linearly with the size of the system. However, because of other factors such as the calculation of long-range electrostatics, in practice the computational effort required when using this approach scales roughly as *N*^1.6^, where *N* is the size of the system.

In addition to being faster than the equivalent matrix-algebra technique, the MOZYME technique is computationally more robust when solving the SCF equations. This is a consequence of an increased HOMO–LUMO gap when LMOs are used (see the next section); as a result, the individual LMOs are less polarizable than the individual CMOs and are therefore less prone to charge oscillation and other instabilities.

An unfortunate side effect of the reduced polarizability is that sometimes the SCF field generated by MOZYME converges to an excited electronic state instead of to the ground state; this occurs most frequently in gas-phase systems that might be assumed—incorrectly—to have highly charged groups. For example, in structures involving multiple –NH_3_ and –COO groups [26], the Lewis structure approach used to start the SCF procedure in MOZYME always generates ionized sites, giving rise to zwitterionic sites. If the lowest-energy SCF electronic structure involved some or all of the charges canceling out, so that the system contained –NH_3_ and –COO instead of –NH_3_^+^ and –COO^−^, then, when the MOZYME SCF is formed, the resulting electronic structure might not be that of the ground state.

The origin of this fault can be understood in terms of the properties of LMOs. At the start of the SCF procedure, the charges on the various atoms would be those assigned by the Lewis structure. If a pair of charges generated by the Lewis structure should not be present in the SCF ground state, then, during the SCF procedure, the LMOs representing these charges would need to migrate from one atom to the next as they moved towards each other under the influence of electrostatic attraction. This would obviously involve a high-energy intermediate if a migrating LMO moved onto a group that was not easily ionized, e.g., a –CH_2_– group, and migration of the LMOs would therefore be inhibited.

In the CMO methods in MOPAC, the starting density matrix is constructed assuming that all atoms are neutral. Because of this assumption, CMO methods would naturally result in the SCF procedure converging to the neutral species if that species has the lower heat of formation (Δ*H*_f_). Conversely, if the zwitterionic form had the lower Δ*H*_f_, then, because the CMOs are naturally delocalized over many atoms and are thus more easily polarized, the SCF procedure would normally converge to the ionized form.

Whenever this occurs, using an implicit solvent in a MOZYME calculation results in a large decrease in the Δ*H*_f_ due to ion–solvent interactions. When CMO methods are used, the energy change on solvation would be equal to that for a MOZYME calculation reduced by the amount of energy required to convert the system from the neutral to the ionized form. Problems of this type are highly artificial in that they only occur in unrealistic gas-phase systems. Indeed, such systems are so unrealistic that no conventional Lewis structure can be written for them, and, if they were to be synthesized, they would almost certainly be highly unstable. The simple expedient of making the system more realistic by using implicit solvation always results in both the LMO and the CMO methods producing the correct ground state.

The other situation where the Lewis structure model fails occurs in large graphitic structures, such as irregular graphene flakes in which hydrogen atoms are attached to the edge atoms. Any mistake made during the construction of the Lewis structure could result in the formation of one or more pairs of charges in a system that should have no formal charges. If these faults were not corrected by using the appropriate keywords to modify the default Lewis structure, the resulting SCF would likely be incorrect.

### Localized molecular orbital energy levels

Because the CMO and LMO sets are related by a unitary transform, the sum of the occupied-set energy levels is the same in both cases, but the individual localized and canonical molecular orbital energy levels are different, the most important difference being that the LMO energy levels span a smaller range than that spanned by the CMOs. This is a direct consequence of the eigenvector nature of CMOs: all interaction energies involving pairs of CMOs are zero. Any unitary transform of the CMO set would introduce nonzero interaction energies between pairs of MOs, and the presence of such interactions would necessarily reduce the difference between the MO energy levels. In the case of the CMO with the lowest energy, any nonzero interaction energy introduced by a unitary transform would cause its energy to increase. A similar effect would take place with the HOMO, where any nonzero interactions introduced by a unitary transform would cause its energy to decrease. When the LMOs and CMOs are similar, the reduction in the range of energies in the LMOs would be small. At the other extreme, exemplified by cubic nonmetallic allotropes such as diamond and cubic binary compounds such as boron nitride, the reduction would be complete. In those systems, the LMOs would all have exactly the same energy, and each LMO would represent a single covalent chemical bond.

A similar set of conditions applies to the virtual or unoccupied CMOs and LMOs in that the range of energies spanned by the unoccupied LMOs is smaller than the corresponding range for the equivalent CMOs, and the LMO LUMO energy would be more positive than that of the CMO LUMO. A direct consequence of these changes is that the LMO HOMO–LUMO energy gap is always larger than the corresponding CMO gap. This increase is very important in that it reduces the polarizability of the LMOs, which, in turn, increases the stability of the SCF procedure.

### Localized molecular orbital sizes

Although localized molecular orbitals have some intensity (the square of the wavefunction) on all the atoms, *A*, in a system, within any specific LMO, ${\psi}_i={\sum}_A{\sum}_{\lambda \in A}{c}_{\lambda i}{\phi}_{\lambda }$, almost all of the intensity is located on only a few atoms. A quantitative measure, *C*_*i*_, of how localized each LMO is can be constructed from these intensities using ${C}_i={\left\langle ii| ii\right\rangle}^{-1}={\left({\sum}_A{\left({\sum}_{\lambda \in A}{c_{\lambda i}}^2\right)}^2\right)}^{-1}$. This quantity is of course noninteger, so, in order to avoid referring to fractional atoms, the size of a LMO should be interpreted as the number of centers involved. For lone pairs, where most of the LMO is concentrated on one atom, *C*_*i*_ is approximately 1.0; for simple two-center single, double, and triple bonds, its value is 2.0; and for delocalized π bonds, *C*_*i*_ has values of 2.5 or higher.

## Relationships between canonical molecular orbitals and localized molecular orbitals

### Converting CMOs into LMOs

Various methods of converting CMOs into LMOs have been proposed, all of which involve performing a unitary transformation. In the Boys technique [27, 28], the geometric size of the LMO is minimized; in the Edmiston–Ruedenberg [29] approach, the sum over all LMOs of the electrostatic self-repulsion is maximized; and in von Niessen’s method [30], the electrostatic repulsion between pairs of LMOs is minimized. The Edmiston–Ruedenberg approach is thus essentially similar to that of von Niessen. This relationship can easily be understood in terms of electron repulsion integrals involving LMOs *ψ*_*i*_ and *ψ*_*j*_, as shown in Eq. 1, where *r* = ∣*r*_1_ − *r*_2_∣:

1 $$ \left\langle ii| jj\right\rangle =\iint {\psi}_i\left({r}_1\right){\psi}_i\left({r}_1\right)\left(\frac{1}{r}\right){\psi}_j\left({r}_2\right){\psi}_j\left({r}_2\right)\mathrm{d}{r}_1\mathrm{d}{r}_2. $$

In the Edmiston–Ruedenberg localization method, the sum of all self-repulsion integrals, ${\sum}_i\left\langle ii| ii\right\rangle,$is maximized, and in von Niessen’s localization method, the sum of all integrals involving different LMOs, ${\sum}_{i<j}\left\langle ii| jj\right\rangle,$ is minimized. Because the sum ${\sum}_{ij}\left\langle ii| jj\right\rangle$is a constant, it follows that these two localization methods are complementary.

A second, completely different, approach [31] involves a Cholesky decomposition of the density matrix. This very recent technique is particularly useful because it is efficient, being noniterative, and versatile, in that it does not require initial CMOs. These advantages might justify using this technique in semiempirical methods, but this possibility does not appear to have been explored yet.

When ab initio methods are used, evaluating the two electron integrals is tedious [32]. An alternative approach, useful in semiempirical methods, involves replacing the operator (1/*r*) in Eq. 1 with unity and then calculating the overlap of the two resulting charge clouds. One consequence of the zero differential overlap approximation in NDDO semiempirical methods is that atomic orbital overlap integrals are neglected in the normalization of MOs, so integrals of the type 〈*ii*| *jj*〉 can be expressed as simple sums of products of atom orbital coefficients. This approximation greatly simplifies von Niessen’s localization procedure and results in a very efficient method for generating an optimized set of LMOs [33].

In some systems, the LMOs involved in constructing double and triple bonds are not always uniquely defined. This can occur when the atoms in a set of LMOs are the same and the number of centers in the LMOs are the same (usually about 2.0). For convenience, a set of LMOs that satisfy these conditions will be referred to here as a degenerate set. Within each degenerate set, the sum of the energies of the LMOs is uniquely defined but the individual component LMO energies are still ill-defined. A similar situation occurs in systems where two or three lone pairs are present on an atom. In all such cases, a simple unitary rotation of the two or three LMOs involved can be used to generate uniquely defined LMOs. For convenience, the following description will refer only to bonds, but it should be understood as also being applicable to lone pairs. A small (size 2 or 3) secular determinant is constructed using the LMO energies *ϵ*_*ii*_ and the cross-terms *ϵ*_*ij*_, representing the interaction energy of the LMOs *ψ*_*i*_ and *ψ*_*j*_. When this determinant is diagonalized, the result is a set of eigenvectors which can then be used as the unitary matrix for rotating the LMOs. Because the secular determinant is energy-based, the resulting LMOs always involve one LMO that has σ character and one (in the case of a double bond) or two (in the case of a triple bond) that have π character. When a triple bond is present, the two LMOs may be degenerate in energy, so their orientation would still be ill-defined. However, this could only occur when there is rotational symmetry about the diatomic axis, so their energies, degrees of localization, and atoms involved would all be the same, meaning that the resulting LMOs would always be equivalent by definition.

An alternative representation of these LMOs can be generated by performing another simple unitary transformation to form a set of new LMOs, each of which has the same amount of the original bond. The transform to convert a double bond involving LMOs *ψ*_σ_ and *ψ*_π_ into new LMOs *ψ*_1_ and *ψ*_2_ is shown in Eq. 2:

2 $$ {\psi}_1={2}^{-1/2}\left({\psi}_{\upsigma}+{\psi}_{\uppi}\right),{\psi}_2={2}^{-1/2}\left({\psi}_{\upsigma}-{\psi}_{\uppi}\right). $$

No unique transform exists for a triple bond involving LMOs *ψ*_σ_, *ψ*_π*x*_, and *ψ*_π*y*_, but, since the new LMOs are essentially degenerate and, as just noted, the precise orientation of the resulting three hybrid LMOs about the diatomic axis can never be important, this lack of uniqueness is once again unimportant and any unitary transform that results in an equal contribution of *ψ*_σ_ to each of the hybrid LMOs can be used. An example of a simple unitary transform that will generate three equivalent bonds is shown in Eq. 3.

3 $$ {\displaystyle \begin{array}{l}{\psi}_1=\left({3}^{-1/2}{\psi}_{\sigma }+{6}^{-1/2}{\psi}_{\pi x}-{2}^{-1/2}{\psi}_{\pi y}\right)\\ {}{\psi}_2=\left({3}^{-1/2}{\psi}_{\sigma }+{6}^{-1/2}{\psi}_{\pi x}+{2}^{-1/2}{\psi}_{\pi y}\right)\\ {}{\psi}_3=\left({3}^{-1/2}{\psi}_{\sigma }-{\left(\frac{3}{2}\right)}^{-1/2}{\psi}_{\pi x}\right)\end{array}} $$

When these sets of equivalent LMOs occur in double [34] and triple bonds, they are commonly referred to as “banana bonds” [35].

Similar transformations can be performed on LMOs that represent lone pairs on an atom. When two lone pairs are present, a transform of the type shown in Eq. 2 would be used, and the resulting hybrid LMOs are commonly referred to as “rabbit ears.” When three lone pairs are present, as in the fluorine atom in CH_3_F, a transform of the type shown in Eq. 3 would be used. In the absence of any external perturbations, the energies and shapes of the resulting set of two or three LMOs would the same.

### Converting LMOs into CMOs

Given that both LMOs and CMOs give rise to the same density matrix and therefore to the same Fock matrix, in practice the simplest and most rapid way to convert LMOs into CMOs is to construct the Fock matrix over atomic orbitals from the LMOs. Diagonalization of that matrix then gives the CMOs.

## Examples of the use of LMOs to describe chemical systems

Seven worked examples will be presented to illustrate the use of LMOs to model chemical systems. All simulations were modeled within MOPAC using the semiempirical method PM7 [23]; PM7 is the default method in MOPAC2016 [36], but similar results are obtained if any of the other modern semiempirical methods are used. Charged species almost always exist in the condensed phase, so, in order to make the model more realistic, the COSMO dielectric screening method [37] was used to mimic solvation effects when these species were modeled. Because the systems involved were all very small, the SCF equations were solved using conventional matrix algebra, and the resulting CMOs were then converted to LMOs using the NDDO localization method [33].

### Butadiene

1,3-Butadiene is frequently used as an example of a small linear polyene whose π-electron system is stabilized by delocalization. One explanation for how delocalization gives rise to stabilization is given by Hückel theory [6]. When applied to butadiene, Hückel theory predicts that the delocalization energy, in units of the interaction energy of a simple *p*-*p* π bond, *β*, would amount to $\left(2\sqrt{5}-4\right)$*β* = 0.472*β*. Modern semiempirical methods also reproduce this delocalization, as shown in the π-CMO coefficients presented in Table 1, where, as expected, both π CMOs are delocalized almost equally over all four carbon atoms. But, while this result suggests that it is the delocalization that causes the extra stabilization, when the CMOs were relocalized, the number of centers spanned decreased to 2.07, essentially the same as that of a simple unconjugated π bond. That delocalization stabilization is a real observable phenomenon is incontrovertible, but equally the attribution of the stabilization to simple delocalization of the π MOs over more atoms cannot be correct, given that the CMOS can be relocalized to (almost) ethylenic bonds without any loss of stabilization energy.

**Table 1:** π Molecular orbitals for 1,3-butadiene

	PM7 Canonical MOs		PM7 Localized MOs		Hückel Localized MOs	
	1au	1bg	π-1	π-2	π-1	π-2
Energy level	−12.126 eV	−9.613 eV	−10.870 eV	−10.870 eV	1.118β	1.118β
No. of centers	3.775	3.712	2.070	2.070	2.215	2.215
Atom	Coefficients		Coefficients		Coefficients	
1	0.4345	0.5653	0.7070	−0.0925	0.6887	−0.1626
2	0.5578	0.4248	0.6948	0.0941	0.6887	0.1626
3	0.5578	−0.4248	0.0941	0.6948	0.1626	0.6887
4	0.4345	−0.5653	−0.0925	0.7070	−0.1626	0.6887

An alternative explanation might be that even a small degree of π delocalization has a profound stabilizing effect. When the secular determinant is diagonalized, the MO coefficients are automatically optimized so that the energy is at a minimum, a condition that exists in the LMO coefficients in Table 1. An examination of the π-1 coefficients shows that the coefficient on atom 3 has the same sign as those on atoms 1 and 2. Delocalization of this type, where the lobes of a π MO expand onto a nearby atom, could be viewed from a quantum-mechanical perspective as increasing the wavelength of the wavefunction, and this would then be responsible for the decrease in its energy. This would be the chemical equivalent of increasing the size of the box in the quantum physics exercise of the “particle in a box,” causing the eigenvalues to decrease in energy. Delocalization of this type manifests itself in other theoretical methods ranging from the Hückel model, also shown in Table 1, to higher-level methods such as Hartree–Fock and density functional theory ab initio methods. In all the cases examined, the π LMOs were qualitatively the same as those for PM7.

An unacceptable consequence of both π-LMO wavefunctions having a positive coefficient on three of the atoms is that, if the coefficient of each wavefunction on the fourth atom were to be zero, the π LMOs would have a small positive overlap and the orthogonality of the MOs would be destroyed. To avoid this, both wavefunctions are required to have a small negative coefficient on the remaining atom.

The conventional explanation for the experimentally-observed lowering of a compound’s heat of formation when a conjugated π-system is present is that the wavefunctions for the π electrons are delocalized, and these delocalized π electrons generate the resonance stabilization that in turn lowers the energy of the system. This description is rooted in the concept of CMOs, and leads naturally and logically to mental constructs such as resonance structures and aromatic stabilization. An alternative explanation based on LMOs would be that the available space for the π electrons is small in isolated double bonds, but the space is increased when delocalization resulting from conjugation is present. In but-2-ene, for example, 99.76% of the π LMO is located in the C2–C3 bond, whereas only 98.26% is located in the C1–C2 bond in but-1,3-diene. The delocalization stabilization in but-1,2-diene would then be attributed to this very small (1.5%) difference.

It is important at this point to reiterate that neither CMOs nor LMOs actually exist. That is, although CMOs have been used in theoretical chemistry for almost a century and have been widely used to explain π-electron stabilization, they are no more real than LMOs. At the same time, although LMOs have a seductive appeal due to their simplicity (obviating as they do entire CMO concepts such as resonance stabilization), they, too, are no more real than CMOs.

### Water

The water molecule provides a good example of a system that has two lone pairs on a single atom. Each molecule has eight valence electrons, represented in the Lewis structure by single bonds between the oxygen atom and the hydrogen atoms and by two lone pairs on the oxygen atom. All self-consistent field methods can reproduce the bonds and lone pairs.

Water belongs to the symmetry point group C_2v_. In CMO form, one of the lone pairs on the oxygen atom belongs to the irreducible representation b_1_, otherwise known as π symmetry, in that it is antisymmetric to reflection through the plane of symmetry. Only one MO of this symmetry is present, so it represents a pure lone pair. The other lone pair MO is of representation a_1_, but as this is the same as one of the O–H bonding orbitals, the two MOs can—and do—mix. As a result of this mixing, the second CMO lone pair MO, although recognizable as a lone pair, cannot not be regarded as a pure lone pair.

**Fig. 1:**
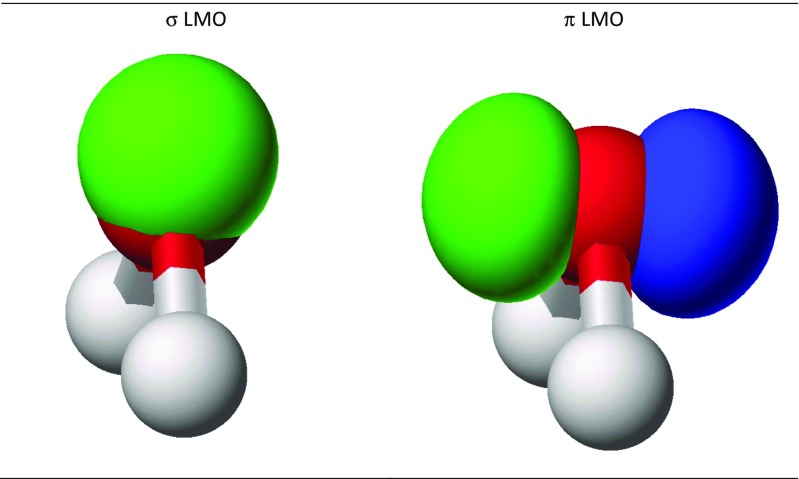
Localized σ and π lone-pair molecular orbitals on the oxygen atom in water

When the CMO set is localized, the three σ MOs, two of representation a_1_ and one of b_2_, resolve into two O–H bonding LMOs and a pure σ lone pair (Fig. 1, left). This is accompanied by an expected decrease in the energy of the σ lone pair, as shown in Table 2. The starting π lone pair MO was already completely localized, so it would remain unaffected by localization.

**Table 2:** Molecular orbital energy levels for the lone pairs in water

MO type	Lone pair 1		Lone pair 2	
	Symmetry	Energy (eV)	Symmetry	Energy (eV)
CMO	a_1_ -σ	−14.223	b_1_ -π	−12.112
LMO using σ and π orbitals	σ	−19.954	π	−12.112
LMO using rabbit ears	Left rabbit ear	−16.033	Right rabbit ear	−16.033

The oxygen atom in water is unusual in that the CMOs for most atoms with two lone pairs cannot be resolved into 100% pure lone pairs. That the π lone pair is completely localized can be understood given the fact that there is only one π atomic orbital and therefore any mixing of MOs is impossible. The reason why the σ lone pair should also be completely localized is not so obvious. One explanation is that, by definition, the two O–H LMOs must be orthogonal, and also by definition the σ lone-pair LMO must be oriented along the C2 axis; therefore, in order for the σ lone pair to be orthogonal to both O–H MOs, it cannot have any intensity on either hydrogen atom, so it is constrained to be 100% localized on the oxygen atom.

The pure lone pairs on water are a consequence of the highly limited basis set used in semiempirical methods. If a larger basis set had been used, the lone pairs would have had some intensity on the hydrogen atoms.

### Diamond

The electronic structure of diamond provides a dramatic example of how the LMO and CMO descriptions differ. CMO descriptions of crystalline solids such as diamond allow the electronic band structure within the Brillouin zone to be generated. Brillouin zones contain a wealth of detail, such as k-space coordinates, e.g., the symmetry points Γ, X, and K have the coordinates (0.0, 0.0, 0.0), (0.5, 0.0, 0.0), and (0.25, 0.25, 0.25), respectively; the eigenfunctions at various points within the Brillouin zone have specific Little group [38] symmetry properties; and the energy band structure can provide information on the electron and positron effective masses used to predict electrical conductivity, and can be used to calculate the electromagnetic properties of the solid. In short, Brillouin zone descriptions of solids are extensive, well understood, and are of great use for predicting physical properties.

By definition, each solid-state CMO extends over an infinite number of atoms and has an infinitesimal intensity on any one atom. This feature alone makes the CMO description of the chemical structure of solids difficult to interpret. In contrast, the LMO description of diamond consists of a large number of simple two-center molecular orbitals, one for each carbon–carbon bond in the system being modeled, and, since all these bonds are equivalent, all the LMOs have the same energy but none of the elegant k-space band structure resulting from using CMOs is present. In diamond, the most important advantage of the LMOs over CMOs is that they provide a measure of the simple covalent carbon–carbon bond energy.

### Halite

In contrast to diamond, where all the bonds between atoms are completely covalent and have no polarization, the interactions between the atoms in a sodium chloride crystal are almost entirely electrostatic, as a result of the atoms being highly ionized. This ionization is reflected in the CMOs, where most of the wavefunction is located on the chloride anion.

The LMOs are similar to the CMOs in that the intensity of the wavefunctions is almost exclusively on the chloride anions, but in contrast to the CMOs, where each MO has the same intensity on every chloride, each LMO presents a high intensity on precisely one chloride ion and a much smaller intensity on the adjacent sodium cations. Localization of the CMOs results in LMOs that have almost pure *s* or *p* character. Hybridization of these LMOs to form four equivalent lone pairs that correspond to the Lewis dot pattern for the chloride ion would be possible, but, since the covalent contribution to bonding is already very small, such an operation would not produce MOs that would provide any more information than could be obtained from unhybridized LMOs.

### Graphite

**Fig. 2:**
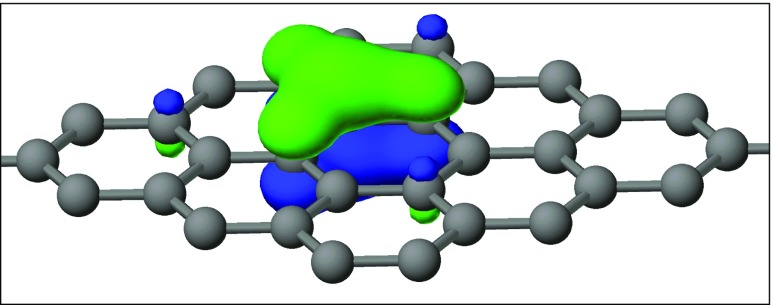
Localized π molecular orbital in graphite

Graphite presents another extreme type of bonding: the extreme delocalization of its π electrons. To investigate the properties of LMOs in graphite, a solid-state calculation using Born–von Kármán [39] periodic boundary conditions was performed on a system of 128 carbon atoms. This produced a set of 192 carbon–carbon σ LMOs and 64 π LMOs. All the σ LMOs were identical in shape and involved 2.03 centers; that is, they were all two-center MOs. As expected, these MOs all had the same energy of −21.25 eV. All the π LMOs were identical and involved 3.22 centers, i.e., they were three-center MOs, and had an energy of −12.55 eV. Each π LMO had threefold symmetry with 50% of the LMO intensity on one atom, 14% on each of three atoms attached to the center atom, and 1.9% on each of three atoms *para* to the central atom. The remaining 2.3% of the LMO was distributed over more distant atoms. An example is shown in Fig. 2, where the green and blue regions indicate positive and negative lobes in the LMO, respectively. A nodal plane exists at each atom *meta* to the central atom. This divides the LMO into a set of four atoms consisting of a central atom and the three atoms that are attached to it, and a set of three atoms that are *para* to the central atom. Each π LMO is centered at the *meta* position of the adjacent π LMOs.

An idealized LMO involving only the atoms mentioned would have 50% of its intensity on the central atom, $$ 13\raisebox{1ex}{$1$}\!\left/ \!\raisebox{-1ex}{$3$}\right.\% $$  $$ 13\raisebox{1ex}{$1$}\!\left/ \!\raisebox{-1ex}{$3$}\right.\% $$atoms *para* to the central atom. These values would allow the LMO to be orthogonal to the adjacent LMOs, but in this form they would not be orthogonal to more distant LMOs. Extended tails are necessary to achieve orthogonality between all the π LMOs, and it is these tails that account for the differences between the calculated and idealized LMOs.

That pairs of adjacent idealized LMOs are orthogonal can readily be demonstrated by selecting one LMO and a second LMO centered on any atom in the *meta* position relative to the central atom of the first LMO and then calculating the overlap given that, at the NDDO level of approximation [5], the integrals for two different atomic orbitals *ϕ*_*λ*_ and *ϕ*_*σ*_ are ∫*ϕ*_*λ*_*ϕ*_*λ*_ = 1 and ∫*ϕ*_*λ*_*ϕ*_*σ*_ = 0.

All π LMOs in graphite have the same triangular symmetry, in contrast to the π LMOs in discrete molecules where the π LMOs are centered on two atoms. To investigate why the LMOs have different shapes, the π LMOs for a hexagonal fragment of graphene were calculated. This fragment consisted of 216 carbon atoms with hydrogen atoms attached to the edge atoms to satisfy valence requirements. Even though the system was large enough that the atoms in the middle would be expected to have an almost graphene-like environment, when the LMOs were examined they all had the same general shape as those in aromatic systems; that is, they had a significant intensity on two atoms and a much smaller intensity on the four adjacent atoms.

Further examination of the individual LMOs revealed a possible reason for the difference between the LMOs in molecules and in graphene. All the π LMOs at the edge of the graphene flake were clearly similar to those for simple aromatic compounds and had an energy of −11.3 eV. On moving towards the center of the flake, the LMO energies dropped to −11.8 eV, and the number of centers increased from ~2.1 to ~3.1, but the qualitative shape of the π LMOs remained unchanged. All the π LMOs in solid graphite had threefold symmetry, the same energy (−12.5 eV), and spanned 3.2 centers. These results can be interpreted as implying that the most stable geometry for LMOs in an extended π-system would have threefold symmetry, and that the shapes of π LMOs in discrete compounds are a result of the strong influence of the atoms at the edges.

### Aromatic systems

Delocalized π-systems in aromatic compounds give rise to a special type of stabilization. A good description of this important phenomenon, together with various aspects of the experimental and theoretical issues involved, is provided in a recent review [40], and no further elaboration need be given here. These π-systems form an important exception to the normal Lewis structure convention, and to indicate their presence and to emphasize the fact that these bonds are unconventional, aromatic rings are usually written with either a dotted or a solid line instead of the Lewis alternating single and double lines. Addressing the question of how these extended π-systems could be represented using LMOs is therefore of interest.

**Fig. 3:**
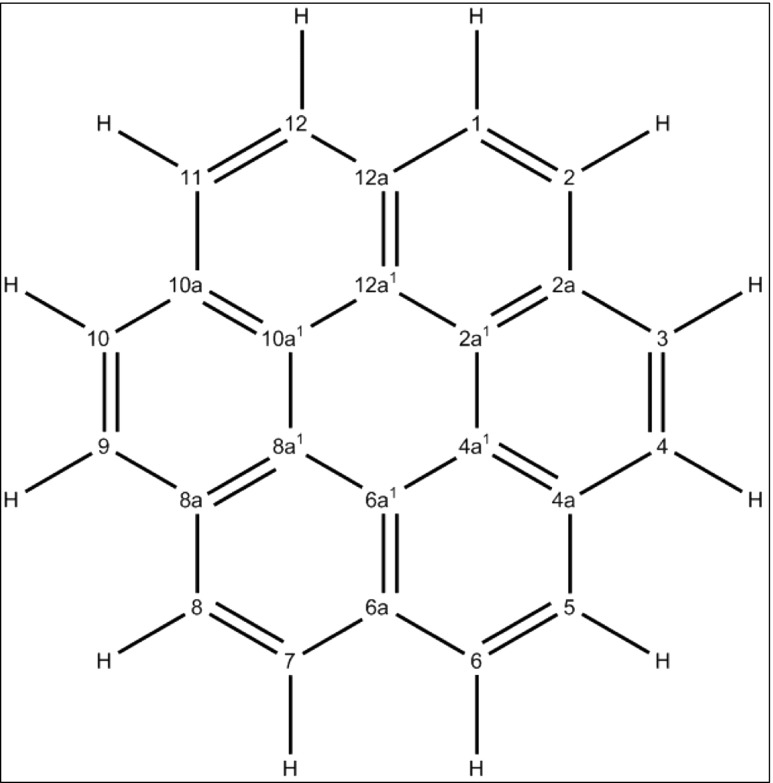
Coronene, C_24_H_12_, showing the numbering system and a Lewis structure

Coronene, a hydrocarbon with an extended delocalized π-system, provides a good test case. Its numbering system and one of the possible Lewis structures is shown in Fig. 3, and a description of its LMOs is given in Table 3. Because of its high symmetry, only seven different types of LMOs are present: five σ bonds and two π bonds. As expected, the 30 C–C σ bonds are the most stable, followed by the 12 C–H σ bonds, and then the 12 π bonds. Also as expected, all of the σ bonds are two-center LMOs; that is, normal Lewis single bonds. On the other hand, the π bonds form two distinct groups. LMOs in the set with an energy level of − 11.13 eV were somewhat localized, spanning 2.4 atoms, and gave rise to strong bond alternation, the bond order for the 1–2 bond being 0.46 greater than that of the 1–12a bond, as shown in Table 4. The other set, at −11.35 eV, consisted of more delocalized LMOs, and gave rise to smaller bond alternation. These LMOs present high intensity on bonds of type 2a–2a^1^, and a lower but still significant intensity on the four adjacent atoms.

**Table 3:** Localized molecular orbitals in coronene

LMO energy level	Description	Type	No. of centers	Degeneracy
−11.13	C1–C2	π	2.43	6
−11.35	C2a–C2a^1^	π	2.99	6
−17.64	C–H	σ	1.97	12
−19.78	C1–C12a	σ	2.02	12
−19.93	C2a^1^–C4a^1^	σ	2.03	6
−20.31	C2a–C2a^1^	σ	2.02	6
−20.60	C1–C2	σ	2.01	6

See Fig. 3 for numbering system

**Table 4:** Bond orders and bond lengths in coronene

Atom pair	Bond order	Bond length from PM7 (Å)	Bond length from [59] (Å)
1–2	1.67	1.367	1.366
2a–2a^1^	1.40	1.403	1.419
1–12a	1.21	1.426	1.416
2a^1^–4a^1^	1.20	1.427	1.422
1–H	0.96	1.089	1.082

All 12 π bonds are delocalized as expected, but because the degree of delocalization is small they could still be easily mapped onto the equivalent Lewis double bonds.

When all the C–C bond lengths were set to be equal, no significant change was found in either the LMOs or the bond orders. This excludes the possibility that the change in bond order was caused by the differing C–C bond lengths, and gives rise to the inescapable conclusion that the differing C–C bond orders in coronene, and therefore the differing C–C bond lengths (see Table 4), are a direct result of the nature of the two sets of π bonds.

### The S_N_2 reaction

A particularly useful application of LMOs is to assist in describing the processes that occur in simple chemical reactions such as the S_N_2 reaction. In a typical organic S_N_2 reaction, an incoming nucleophile reacts with an aliphatic carbon atom that is bonded to an ionizable ligand to form a transient pentacoordinate transition state, which then decomposes when the original ligand is expelled and the attacking nucleophile completes the formation of a normal covalent bond. When the carbon atom is chiral and inversion of the configuration occurs, reactions of this type are described as involving a Walden inversion [41, 42].

**Fig. 4:**
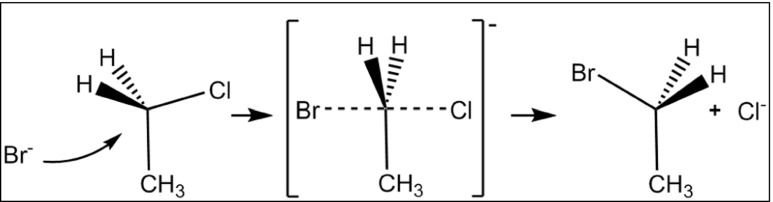
S_N_2 reaction of Br^−^ + CH_3_CH_2_Cl → CH_3_CH_2_Br + Cl^−^

The various changes that occur in a S_N_2 reaction can be illustrated using the reaction shown in Fig. 4. In this reaction, an isolated bromide anion reacts with chloroethane to give bromoethane and an isolated chloride anion. The starting geometry for modeling the S_N_2 reaction was the transition-state geometry generated using the SADDLE [43] method and optimized using Baker’s EigenFollowing technique [44]. Perturbations were then made to this geometry, displacing it a small distance along the reaction coordinate normal mode in the directions of the reactants and products to generate two systems, one on each side of the transition state. These displacements resulted in small forces being generated on all the atoms in the system and allowed the intrinsic reaction coordinate (IRC) [45] to be mapped out. The IRC was calculated using mass-weighted Cartesian coordinates, and expressed in Cartesian (i.e., non-mass-weighted) coordinates relative to the transition-state geometry. This choice of units allowed the reaction coordinate axis to be represented simply in angstroms.

At the start of the IRC, the small magnitude of the forces involved produced some computational artifacts in the trajectory. These were eliminated by replacing the calculated points with a low-order polynomial trend line.

**Fig. 5:**
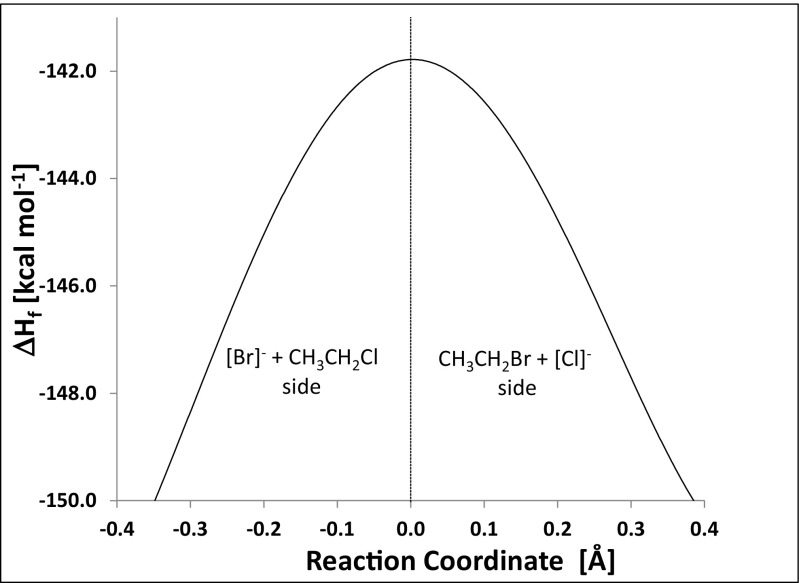
Intrinsic reaction coordinate for the S_N_2 reaction Br^−^ + CH_3_CH_2_Cl → CH_3_CH_2_Br + Cl^−^. The origin of the reaction coordinate is the geometry at the top of the reaction barrier. Negative coordinates indicate the reactant side of the barrier and positive coordinates indicate the product side

**Fig. 6:**
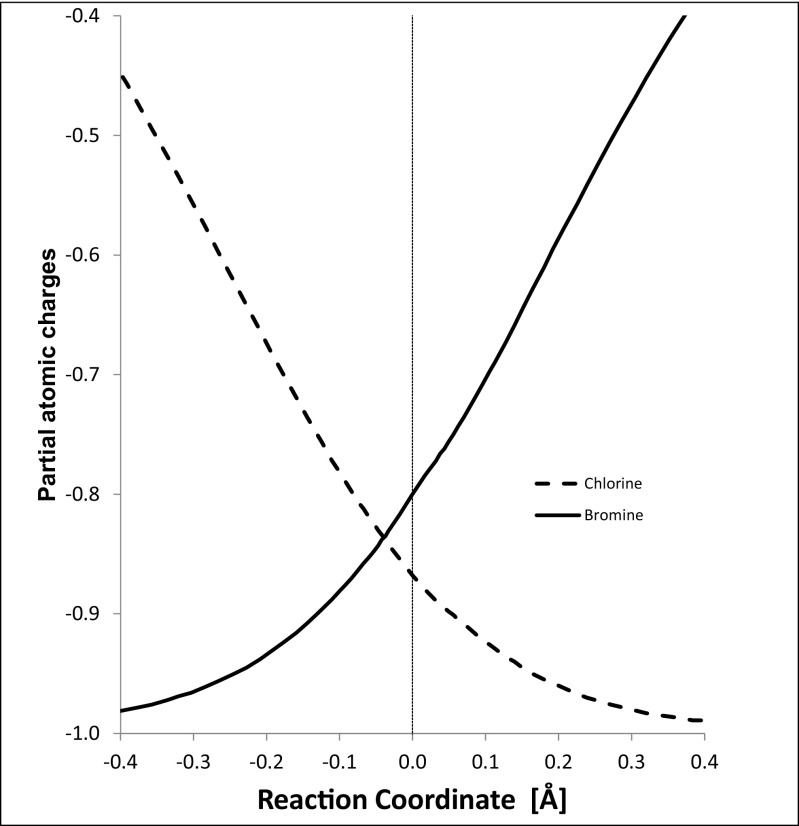
Partial atomic charges on bromine and chlorine

Both the CMO and the LMO descriptions of the processes that occur during the reaction are identical for those calculables for which there were equivalent experimental observables. The energy profile along the reaction coordinate for this S_N_2 reaction is shown in Fig. 5. During the reaction, the partial atomic charge on the bromine atom became less negative monotonically as the C–Br covalent bond formed, and the charge on chlorine became more negative monotonically as the C–Cl bond broke, as shown in Fig. 6. Although partial atomic charges are sometimes regarded [46] as being non-observables, the magnitude of the partial atomic charge on an atom could in principle be determined experimentally by applying an electric field to it and measuring the force acting on the atom’s nucleus. Of course, carrying out such an experiment would be extremely difficult, but, since there is no a priori reason that such measurements cannot be made, partial atomic charges should be regarded as observables. In addition, since partial atomic charges result from an integration of the state function followed by a partitioning onto atomic nuclei, they are also physically meaningful observables in a quantum theoretical sense.

**Fig. 7:**
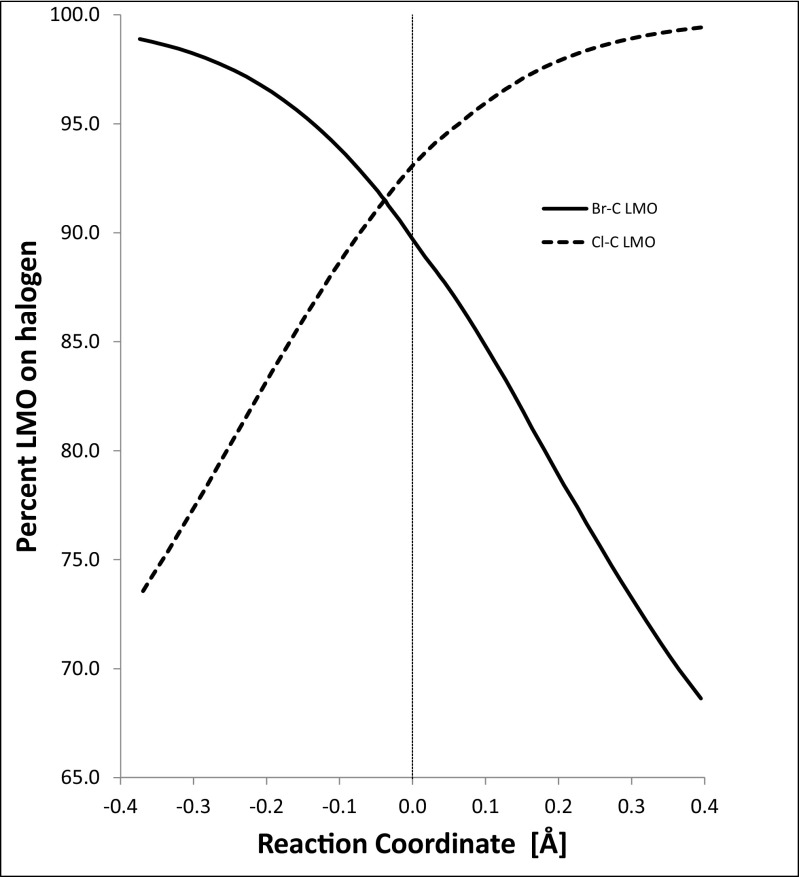
Percentage of the localized molecular orbital on the halogen in halogen–carbon bonds

The utility of LMOs can be illustrated by examining the changes in the Br···C and C···Cl interactions as the reaction proceeds. At the start of the reaction, the Br–C bond would be nonexistent and the LMO representing the Br–C bond would be located entirely on the bromide anion. This corresponds to one of the four conventional Lewis lone pairs on a halide anion. As the reaction proceeds, the percentage of the LMO on the bromine atom decreases (Fig. 7) as the Br–C covalent bond forms, while simultaneously the percentage of the LMO representing the C–Cl bond that is located on the chlorine atom increases as the covalent bond breaks, and a lone pair forms on the chlorine atom.

**Fig. 8:**
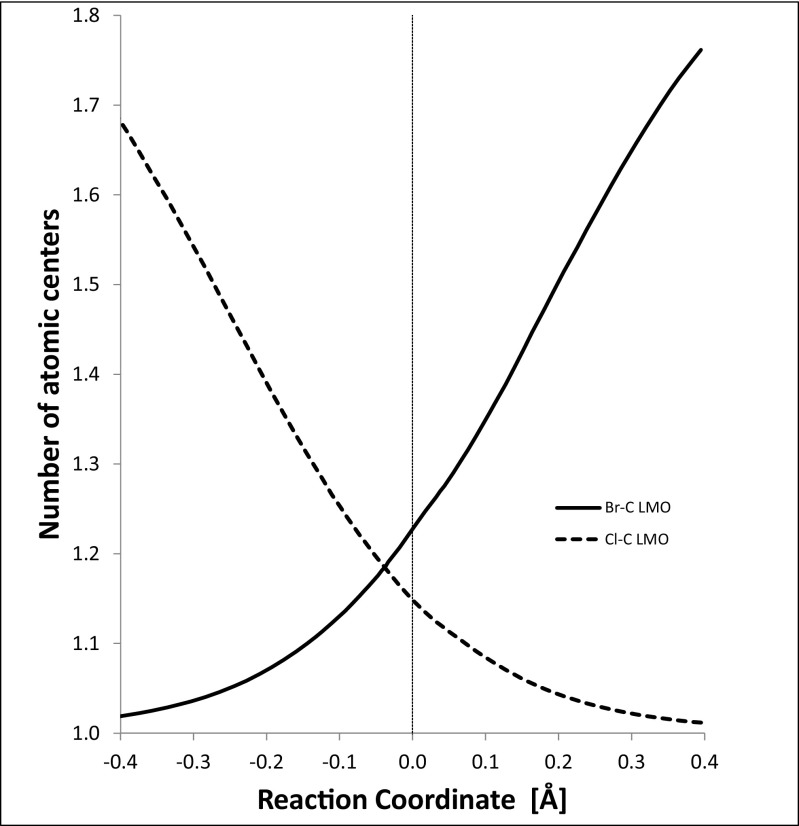
Number of atoms involved in localized molecular orbital bonds

Also, at the start of the reaction, the number of atoms participating in the Br–C LMO is 1.0, reflecting the fact that the LMO corresponds to a lone pair on bromine. As the reaction proceeds (Fig. 8), the number of atoms participating steadily increases due to the formation of a normal Lewis two-center bond. At the same time, the number of atoms participating in the C–Cl LMO decreases as the bond breaks, and the character of the LMO changes to that of a lone pair on chlorine.

**Fig. 9:**
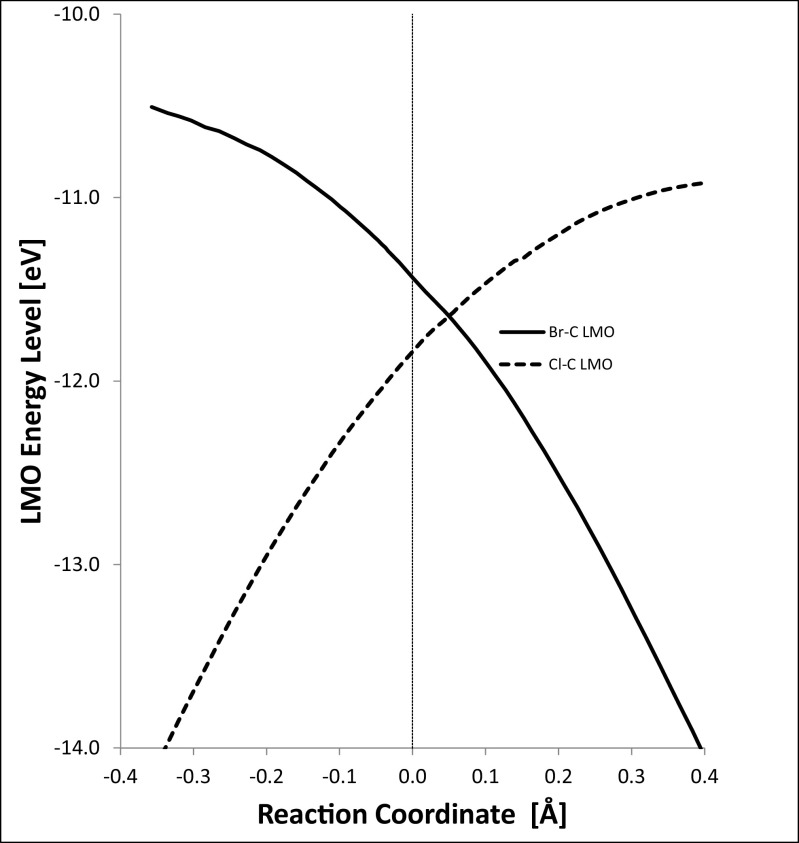
Localized molecular orbital energy levels (eV)

This behavior is also reflected in the changing LMO energy levels, as shown in Fig. 9. Each of the two LMOs retains its own halide character across the entire reaction. This can be contrasted with the behavior when CMOs are used. In the region where the energy difference between the two CMOs representing the carbon–halogen bonding MOs is small, the character of the CMOs changes smoothly from being mainly carbon and one halogen to being mainly carbon and the other halogen, and, instead of the energy levels crossing as the reaction proceeds, a gap exists between them at all points, in accordance with the requirements of the non-crossing rule [47].

**Fig. 10:**
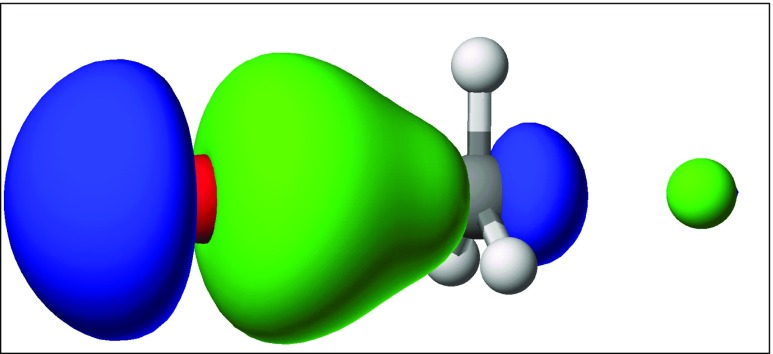
Localized molecular orbital for a Br–C bond at the transition state. Bromine (*red*) forms a σ bond with carbon (*gray*)

**Fig. 11:**
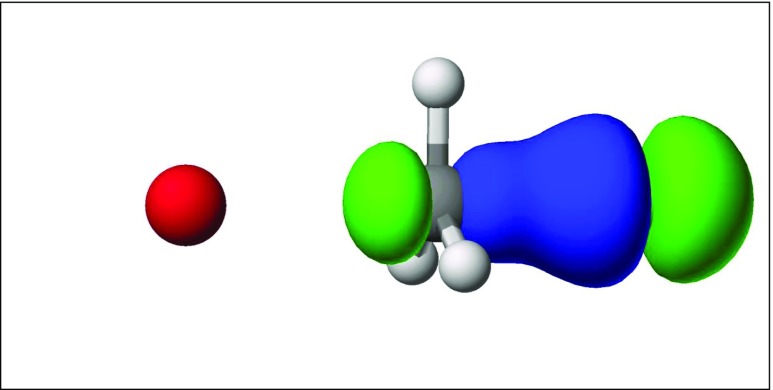
Localized molecular orbital for a C–Cl bond at the transition state. Chlorine, hidden between the green and blue lobes of the C–Cl LMO, forms a bond with carbon (*gray*)

The most interesting point in the reaction profile is the stationary point corresponding to the top of the reaction barrier. There, the carbon atom is pentacoordinate and forms partial covalent bonds with both halogen atoms. Each of these bonds is represented by a LMO: for bromine, this is shown in Fig. 10, and for chlorine in Fig. 11. Thus, from a chemical perspective, the LMOs provide a clear and simple quantitative description of all the changes that occur during the reaction.

## Discussion

### Comparison of canonical and localized molecular orbitals

Lewis structures [24] have been used in chemistry for over a century, and have proven extremely useful for describing chemical bonding in a wide range of chemical systems. In addition, by using curly arrows, Lewis structural elements have been extended to show the movement of electrons and changes in bonding that occur during a chemical reaction. The Lewis structure model of chemistry has thus been a valuable tool for interpreting chemical phenomena.

With the rise of computational chemistry methods, particularly in the past few decades, highly detailed descriptions of electronic phenomena that occur in chemistry have been developed. Most of these descriptions use canonical molecular orbitals, but, while these can provide a quantitative description of electron distributions, the elegance and simplicity of Lewis structures cannot be reproduced easily because most CMOs are delocalized over many atoms.

Both LMOs and CMOs have their own specific advantages, and these will now be described.

#### Advantage of LMOs over CMOs

LMOs can be mapped one-to-one with the various Lewis structural elements of a chemical system, and to that degree they can be said to encapsulate many of the ideas of chemical structure. This mapping is fundamentally different to that used in the conventional computational chemistry representation of chemical structures in terms of bond orders in that a LMO is a two-electron wavefunction that represents a specific Lewis element. Furthermore, because they are derived from a quantum-chemical calculation, each LMO has an associated energy level that can also be associated with a specific Lewis structural element. Prior to the development of LMOs, the concept of assigning an energy to a Lewis bond, a lone pair, or any other structural element did not exist. LMOs thus provide a unique quantity that can be used to describe a chemical structure.

One possible limitation of Lewis structures involves the representation of extended conjugated π-systems. Normal covalent bonds are drawn in Lewis structures using one, two, or three straight lines. This works well for most compounds, but not for aromatic and other compounds that contain an extended conjugated π-system. In such systems, the Lewis structure would consist of an alternating pattern of single and double bonds. This does not capture the nature of delocalized π-systems, and has contributed to a decrease in the use of Lewis structures to describe the molecular orbital structures of systems that have delocalized electrons.

However, an analysis of the structure of LMOs reveals that the degree of delocalization in conjugated π-systems is considerably less than is commonly believed. For example, in coronene, a system containing seven unsaturated hexagonal rings, the most delocalized LMO was found to span only three centers. Even in graphite, a system with an essentially infinite delocalized π-system (one so large that the CMO HOMO–LUMO gap is exactly zero), each LMO spans only 3.22 centers. This small degree of delocalization should not be construed as diminishing the importance and significance of an extended conjugated system. Rather, it should be re-interpreted as an indication that even a small degree of delocalization has a profound influence on chemical behavior.

The influence of the degree of delocalization can be seen in the energies of various LMOs. Thus, in coronene, the more localized π LMOs (Table 3) all had an energy of −11.13 eV. This dropped to −11.35 eV in the more delocalized LMOs, and in graphite the π LMOs all had an energy of −12.55 eV. Given that the difference in delocalization in going from the more delocalized π LMO in coronene (2.99) to the π LMO (3.22) in graphite was only 0.23, the difference in energy, 1.20 eV, could be construed as a dramatic testament to the importance of delocalization.

So, while the Lewis structure for an extended conjugated π-system is incorrect to the degree that it oversimplifies the description of the bonding by completely failing to indicate the presence of delocalized electrons, the corresponding LMO picture represents a large improvement in that it gives rise to molecular orbitals that reproduce the delocalization and the stabilization energy caused by that delocalization.

#### Advantage of CMOs over LMOs

Certain chemical phenomena, particularly those that involve compounds that have conjugated π-electron systems, are more easily understood in terms of CMOs than in terms of LMOs. For example, the likelihood of a Diels–Alder reaction [48] occurring can be predicted using the Hoffman–Woodward rules [49, 50], which depend on frontier molecular orbital (FMO) theory [51], which in turn depend on CMOs. Although in principle these phenomena can be interpreted using LMOs, in practice the CMO description is both simpler and more sound theoretically, and, because there is a vast body of knowledge relating to FMO theory dating back to the original Fukui theory of reactivity [52], there is no obvious reason to advocate developing a new alternative explanation for the mechanism of Diels–Alder reactions based on LMOs.

CMOs provide a simpler description of some physical properties such as the ionization potential, electrical conductivity, and UV-visible spectra. The prediction of these properties depends on the eigenfunction nature of CMOs. For example, Koopmans’ theorem [53] states that the value of the first ionization potential can be predicted as the negative of the energy of the highest occupied CMO. In the case of electrical conductivity, the second derivative of the energy bands with respect to reciprocal space vector k can be used to calculate the electron and hole effective masses, and, when predicting UV-visible spectra, it is much easier to work with state functions using CMOs.

Finally, because CMOs are eigenvectors of the Hamiltonian, they are ideally suited for modeling phenomena that depend on the state of the system. A good example of an experimental quantity that can be expressed in terms of quantum state functions is the photoexcitation spectrum, where a chemical system absorbs a photon and transitions from one state, typically the ground state, to another state. State energies are readily calculable using configuration interaction (CI) methods when CMOs are used. For example, in the construction of the CI matrix for the interaction energies of microstates, if two microstates differ by exactly one MO (i.e., except for MO *ψ*_*i*_ in microstate Ψ_*a*_ and MO *ψ*_*j*_ in microstate Ψ_*b*_, Ψ_*a*_ = Ψ_*b*_), the secular determinant matrix element 〈Ψ_*a*_| *H*| Ψ_*b*_〉 is given as a simple function that includes the integral *ϵ*_*ij*_ = 〈*ψ*_*i*_| *H*| *ψ*_*j*_〉 = ∫ *ψ*_*i*_ *H* *ψ*_*j*_*d*_*v*_. In general, this integral is difficult to calculate, but when a self-consistent field exists and the molecular orbitals are eigenfunctions of the Hamiltonian, all integrals of this type are, by definition, zero. All other terms in the function consist only of phase factors and individual two-electron integrals over CMOs.

In contrast, when LMOs are used and a self-consistent field exists, nonzero integrals of the type *ϵ*_*ij*_ are present, and construction of the CI matrix using LMOs becomes very difficult—so much so that it is computationally more efficient to convert the LMOs into CMOs and then perform the CI calculation using the CMOs.

### Validity of different molecular orbitals

It is important to reiterate that none of the various molecular orbitals used in quantum chemistry are experimental observables. There is a strong temptation to regard certain forms of MOs as having some physical reality, but that is merely wishful thinking. From a quantum theoretical perspective, MOs are not observables, and from an experimental perspective, although certain reactions—particularly those involving delocalized π-systems—can be interpreted in terms of CMOs, these interpretations cannot, and should not, be used as evidence of the CMOs having any real physical existence. Some types of MOs such as Wannier functions [54, 55]—complex eigenfunctions that allow band structures for crystalline solids to be generated—can be related to experimental observables such as band gaps, excitation energies, and electrical conductivity. But all these experimental properties are system properties, and, in all such cases, the properties of the one-electron wavefunctions provide a good approximation to the behavior of the state function; that is, to an observable. However, this relationship does not hold for finite chemical systems, even very large ones. Because of that, the choice of which form of MO to use in chemical systems should depend only on the purpose for which the MOs will be used. Modeling system properties and those chemical properties that depend on specific electronic phenomena such as extended delocalized π-systems is best done using CMOs, whereas LMOs are likely to be more useful for representing purely chemical phenomena, especially those that occur during chemical reactions.

### Localized molecular orbitals from MOZYME calculations

Given that the primary objective of the MOZYME procedure is to solve the self-consistent field equations for chemical systems by using localized molecular orbitals derived from Lewis structures, the resulting SCF LMOs are axiomatically not fully localized. They can, however, be localized by performing a molecular orbital localization procedure. As the starting default SCF LMOs would already be partially localized, this post-SCF re-localization operation is very rapid when compared to localizing a set of SCF CMOs. Thus, for a modified chymotrypsin system based on the PDB entry 8GCH (a medium-sized protein of 3968 atoms and 243 residues), localizing the CMOs from a conventional calculation required 48,768 s, whereas re-localizing the LMOs from a MOZYME calculation required only 350 s. These times can be compared with those for a single MOZYME SCF calculation, 297 s, and that needed to convert the SCF LMOs to CMOs, 1203 s. During this test, the set of LMOs resulting from re-localization of the MOZYME MOs and the set resulting from localization of canonical MOs from a conventional SCF calculation were compared and were confirmed to be equivalent.

### How useful are hybridized localized molecular orbitals?

The case has been made by Clauss et al. [56] (hereafter referred to as P1) that the use of rabbit ear hybrid-based localized molecular orbitals is anachronistic. Some objections to this were raised by Hiberty et al. [57] (hereafter referred to as P2), who defended the idea that no one set of molecular orbitals was intrinsically superior to any other provided they both produce the same electron distribution. In a rebuttal, Clauss et al. [58] (hereafter referred to as P3) agreed with P2 on many points, but reiterated the opinion that concepts such as rabbit ears and valence shell electron pair repulsion (VSEPR) were not useful and that the teaching of them should be discouraged.

Much of the controversy appeared to center on definitions, such as which type of molecular orbital could be defined in the best (in this context the most unambiguous or unique) way. Other issues mentioned in P1, such as fractional molecular orbital occupancy and which sets of MOs best describe delocalization phenomena, are not relevant here. What are relevant are the arguments, assertions, and conclusions regarding the properties of different types of MO models.

Regarding the validity of the different MO models, the following points are assumed to be uncontroversial:

- When any set of occupied MOs is rotated using a unitary transform, the resulting MOs produce the same electron density distribution as that of the unrotated set. That is, from a quantum chemistry perspective, both sets of MOs are equivalent and, because they are non-observables, neither set has any physical meaning.
- In conventional (that is, matrix algebra) SCF methods, when the secular determinant is diagonalized, the resulting MOs are eigenfunctions of the Hamiltonian. These are the CMOs discussed here.
- In systems that have Abelian symmetry (point groups C_1_ to D_2h_), all CMOs are uniquely defined as a consequence of all the eigenvalues being unique. CMOs for systems that belong to non-Abelian point groups can have degenerate eigenvalues, and within each degenerate set the resulting CMOs are not uniquely defined; however, with the exception of point groups I and I_h_, there is always a unitary transform that can convert a degenerate set of CMOs into a set of CMOs that would be uniquely defined.
- When a double or triple bond exists, the associated CMOs are usually split into a σ bond and one or two π bonds. When there are two or three lone pairs on an atom, the associated CMOs usually split into one CMO that has high *s* atomic orbital character and one or two that have mainly *p* atomic orbital character.
- When a set of CMOs is converted into a set of LMOs, no double or triple bonds are present, and no atoms have more than one lone pair, the resulting LMOs are uniquely defined.
- LMOs are sometimes not uniquely defined when double or triple bonds are present. This occurs when the degree of localization is the same for two or three LMOs that participate in a multiple bond: about 2.0. However, degenerate LMOs resulting from the localization of CMOs do tend to retain the character of the CMOs, and so LMOs representing double or triple bonds normally split into MOs that have mainly σ or mainly π character.
- Sometimes LMOs are not uniquely defined when there are two or three lone pairs on an atom. In these cases, the degenerate LMOs resulting from the localization of CMOs tend to split into one LMO that has mainly *s* character and one or two that have mainly *p* character.
- In both previous cases, a simple unitary transform of a degenerate set of LMOs can convert them into a set of well-defined LMOs.
- Degenerate but well-defined sets of LMOs can be converted into a set of hybrid LMOs that have the same, or almost the same, energies. These are the “rabbit ears” and “banana bonds” hybrid molecular orbitals.
- Within any degenerate set of hybrid LMOs, the amount of *s* character in each LMO is roughly the same.

Most of P1 and P3 focus on the pedagogic aspects of different representations of molecular orbital sets. As the author has only a limited understanding of the issues involved, any objections to the points made would be to merely cavil. Equally, and for the same reason, no support or objections can be assigned to the comments in P2 arguing against various pedagogic points in P1.

However, to the degree that molecular orbital theoretical issues are addressed, the author—being familiar with computational chemistry issues—feels qualified to offer an opinion for consideration.

The topic of whether the lone pairs in water can be represented by two “equivalent” rabbit ears was discussed in P1. The authors assert that “Whether one can find some unitary mixture of lone-pair MOs that gives resulting equal-energy orbitals is essentially irrelevant.” The validity of this assertion was questioned in P2, and a rebuttal was given in P3.

**Fig. 12:**
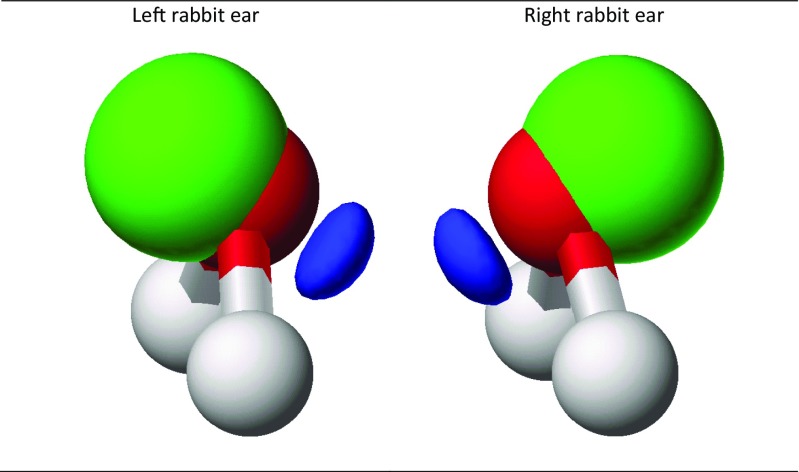
Localized rabbit ear lone-pair molecular orbitals on the oxygen atom in water

Within the program MOPAC, the keyword “RABBIT” can be used to convert a CMO set into a LMO set that differentiates sets of lone pairs on an atom first into σ and π lone pairs and then uses Eq. 2 to re-hybridize these lone pairs into rabbit ears. This operation is straightforward and uncontroversial, as noted in Eqs. 1a and 1b in P2 for atoms that have two lone pairs, such as the oxygen atom in water. When RABBIT was applied to water, the resulting rabbit ears were unambiguously symmetric (Fig. 12) and degenerate in energy (Table 2). When three lone pairs are present, RABBIT would use Eq. 3 to re-hybridize them into three *sp* hybrids.

For more complicated systems, such as enzymes (systems in which hydrogen bonds, and therefore rabbit ear LMOs, are of paramount importance), a cursory inspection of a graphical representation of any rabbit ears structure shows that they are equivalent. As expected, an analysis of the numerical representation of the rabbit ears did reveal small, insignificant differences, but in all cases the energies of the rabbit ears on all atoms were essentially the same.

In P3, the assertion is made that “Whether eigen-orbitals of the 1st-order reduced density operator (Löwdin, 1955) are considered “real” is open to philosophical discussion, but the fact that this operator provides quantitative criteria to determine which of several possible hybrid forms is “best” in describing the actual electron density is indisputable.” This statement was a rebuttal to the assertion in P2 that “one must give up the belief that there exists a unique set of supposedly “real”, or “best”, orbitals.”

Asserting that the reality of eigen-orbitals is a philosophical issue would require reopening the topic of observable versus non-observable. There is no need to do this: as stated repeatedly above, by their nature wavefunctions for electrons are not observables, so they have no physical reality. They are, however, useful for understanding chemical phenomena, and in that sense they are real, but only to the degree that they have pedagogic or interpretive value. Unless some new aspect of this issue arises, this particular point should not be regarded as being in contention.

The assertion that the actual electron density is “best” described by CMOs is clearly incorrect. The correct relationship was given in P1: “... the choice of “MOs” can be rather arbitrary, insofar as any unitary transformation of MOs leads to the same single-determinant wavefunction with no effect on the energy or other observable properties of the system. MOs therefore provide no criterion for which unitarily equivalent set is considered “best,” because all satisfy the full double-occupancy condition.”

On the other hand, it is valid to assert that, depending on the use to which they are ultimately put, one set of MOs is better than another set. Until recently, most quantum chemistry programs generated CMOs, so CMOs became very popular—so popular that there was a tendency to regard them as having a certain reality. This misconception would be an understandable consequence of conflating the profound usefulness of CMOs in interpreting chemical phenomena with the actual phenomena involved. If, instead of being based on CMOs, quantum chemistry programs had evolved from an original method that had been based on LMOs, then it is conceivable that the opposite misconception might have arisen: that, because LMOs are useful for interpreting chemical phenomena, particularly hydrogen bonding and reaction mechanisms, they were in some way the cause of that phenomena.

The fact is, quantum chemistry programs did evolve using CMOs, so whether LMOs would have been better for describing chemical phenomena is now moot. As P1 notes, computational chemistry methods have rendered certain types of LMOs—specifically rabbit ears—an “orbital anachronism.” However, recent developments in quantum chemistry programs have greatly simplified the generation of LMOs, meaning they are now much more accessible for use in modeling.

Concepts such as rabbit ears are of enormous value in chemistry, particularly for understanding biochemical phenomena, but access to them has hitherto been limited by technical constraints. Now that these constraints have been lifted, LMOs are readily available for use in computational chemistry modeling. Hopefully rabbit ears and banana bonds will once again become popular and cease to be an “anachronism.”

## Conclusions

For many decades, little attention has been paid to the properties and uses of localized molecular orbitals; attention has focused instead on canonical molecular orbitals. But LMOs have many useful properties that make them valuable for describing chemical phenomena, in particular:

- All sets of CMOs can be converted into LMOs. This includes systems as different as diamond (purely covalent) and halite (almost completely ionic) and extended π-systems (butadiene and graphite).
- Each LMO in a chemical system can be related to a specific element in a Lewis structure diagram.
- Each LMO has an energy that can be used to describe phenomena that occur in chemical structures. For example, energies can be associated with specific double bonds, in both σ and π form and as banana bonds, and with lone pairs, in both *s* and *p* form and as rabbit ears. Both banana bonds and rabbit ears can be generated by using keywords “BANANA” and “RABBIT” in the program MOPAC.
- Some quantities, in particular the degree of delocalization of a LMO, are of great importance for interpreting chemical phenomena, but these quantities cannot be modeled when CMOs are used.
- Changes in individual components in a chemical reaction can be modeled. In the case of the S_N_2 reaction described here, the changes that occurred in the character and energy of the two pairs of electrons that participated in bond breaking and bond making could be monitored throughout the reaction process.
- Because of their extreme simplicity, LMOs can be used instead of atomic orbitals as the primitive wavefunctions in the construction of secular determinants. This results in a significant reduction in computational effort for large systems and allows much larger systems to be modeled.

The program MOPAC was developed with the objective of it being a useful tool for investigating processes that occur in chemical systems, especially enzyme-catalyzed reaction mechanisms. However, the use of canonical molecular orbitals has for decades limited the usefulness of MOs in describing various chemical phenomena. Now, using keyword requests, a simple and rapid procedure for replacing canonical molecular orbitals with localized molecular orbitals can be performed. The character and energies of specific LMOs can then be used to obtain insight into the processes that occur in enzyme mechanisms.
